# Effect of Wheat Dietary Fiber Particle Size during Digestion *In Vitro* on Bile Acid, Faecal Bacteria and Short-Chain Fatty Acid Content

**DOI:** 10.1007/s11130-016-0537-6

**Published:** 2016-02-29

**Authors:** Krzysztof Dziedzic, Artur Szwengiel, Danuta Górecka, Elżbieta Gujska, Joanna Kaczkowska, Agnieszka Drożdżyńska, Jarosław Walkowiak

**Affiliations:** Department of Pediatric Gastroenterology and Metabolic Diseases, Poznan University of Medical Sciences, Szpitalna 27/33, 60-572 Poznań, Poland; Institute of Food Technology and Plant Origin, Poznan University of Life Sciences, Wojska Polskiego 31, 60-624 Poznań, Poland; Department of Food Service and Catering, Poznan University of Life Sciences, Wojska Polskiego 31, 60-624 Poznań, Poland; Department of Commodity Sciences and Food Analysis, University of Warmia and Mazury in Olsztyn, Plac Cieszyński 1, 10-957 Olsztyn, Poland; Department of Biotechnology and Food Microbiology, Poznan University of Life Sciences, Wojska Polskiego 48, 60-627 Poznań, Poland

**Keywords:** Wheat fibre, *In vitro* digestion, Bile acids, Faecal bacteria, Short-chain fatty acids

## Abstract

The influence of bile acid concentration on the growth of *Bifidobacterium* spp. and *Lactobacillus* spp. bacteria was demonstrated. Exposing these bacteria to the environment containing bile acid salts, and very poor in nutrients, leads to the disappearance of these microorganisms due to the toxic effect of bile acids. A multidimensional analysis of data in the form of principal component analysis indicated that lactic acid bacteria bind bile acids and show antagonistic effect on *E. coli* spp. bacteria. The growth in *E. coli* spp. population was accompanied by a decline in the population of *Bifidobacterium* spp. and *Lactobacillus* spp. with a simultaneous reduction in the concentration of bile acids. This is direct proof of acid binding ability of the tested lactic acid bacteria with respect to cholic acid, lithocholic acid and deoxycholic acid. This research demonstrated that the degree of fineness of wheat dietary fibre does not affect the sorption of bile acids and growth of some bacteria species; however, it has an impact on the profile of synthesized short-chained fatty acids. During the digestion of a very fine wheat fibre fraction (WF 90), an increase in the concentration of propionic and butyric acids, as compared with the wheat fiber fraction of larger particles - WF 500, was observed. Our study suggested that wheat fibre did not affect faecal bacteria growth, however, we observed binding of bile acids by *Bifidobacterium* spp. and *Lactobacillus* spp.

**Electronic supplementary material:**

The online version of this article (doi:10.1007/s11130-016-0537-6) contains supplementary material, which is available to authorized users.

## Introduction

Bile acids play a significant role in lipid metabolism. The importance of conjugated bile salts in facilitating fat absorption has been well established. Many studies have defined which bile salts are bound by commonly used, non-nutritive dietary components [1–5]. Primary bile acids, such as cholic and chenodeoxycholic acids are synthesized in the liver. Next they are secreted into bile after the conjugation of carboxyl group with glycine or taurine, or, in exceptional cases, with other amino acids. After conjugation with glycine or taurine they are secreted into bile and stored in the gall bladder. They are then secreted into the duodenum and play many physiologically important functions, particularly in the regulation of bile secretion and digestion of lipids [6–8].

Complex carbohydrates, which are intrinsically indigestible, are fermented by colonic bacteria to produce short-chain fatty acids (SCFA). It has been documented that these SCFA constitute 3–9 % of human daily caloric intake. Furthermore, colonic bacteria also contribute to the limitation of bile acid active transport in the distal ileum. The processes of intestinal tract conjugation, deconjugation and modification by faecal bacteria lead to the creation of numerous secondary bile acids, such as lithocholic acid (LCA) and deoxycholic acid (DCA). Studies comparing tumour incidences in germ-free and conventional rats suggest that secondary bile acids have a greater promoting activity than the corresponding primary acids [6]. In the human gastrointestinal system, 14 species of *Bifidobacterium* can be found. These bacteria synthesize choloylglycine hydrolase and are capable of bile salt hydrolysis, which also enhances their probiotic properties. Hydrolysis of bile salts is one of the most widely known microbial ways of bile salt biotransformation [6]. In healthy persons 97 % of bile acids are reabsorbed in the terminal ileum, while increased concentration of bile acids can be found in the faeces of patients with ileal disease, resection, or patients ingesting chenodeoxycholic acid for gall stone dissolution [7, 8]. The content of DCA and LCA increases in the colonic contents of humans in response to a high fat diet. The increase in the level of these bile acids presumably reflects increased deposition of bile acids in the gastrointestinal tract in order to emulsify the increased level of dietary fat. The effect of dietary fibre on experimental fat or secondary bile acid binding varies depending on the type of dietary fibre fractions consumed. Wheat bran seems to exert its protective role by decreasing the bile acid concentration in the luminal content of the colon [8]. Because of the postulated relationship between diet, faecal bacteria, bile acids and colon cancer, it seemed desirable to test the processes of bile acid binding, especially secondary bile acid, for different degrees of fineness of wheat fibre, when digested *in vitro* [9, 10]. In adults, approximately 500 mg of cholesterol is transformed into bile acids and in this form is secreted into the gall bladder. Transformation of cholesterol into bile acids is one of the main ways of reducing its blood content. Knowing the role bile acids play in the metabolism of cholesterol, it is essential to learn as much as possible about the process of their biosynthesis and its regulating factors. Dietary fibre is seen as one of those regulators. The role of soluble and insoluble fractions of dietary fibre in binding both primary and secondary bile acids has been documented by many authors [2, 11–13]. The degree of acid binding also depends on the percentage composition of different fibre fractions in the product, and the type of bile acid in question [1]. There are many papers examining the influence of dietary fibre fineness on bile acid binding capacity [14–16], however, no data explaining the bile acid binding capacity of dietary fiber of different degrees of fineness in the proposed *in vitro* research.

For the reasons explained above the goal of this paper was to determine the effect of wheat fibre fineness on bile acid binding ability, the presence of bacteria and the content of short-chain fatty acids when digested *in vitro*.

## Materials and Methods

### Materials

The material for this research consisted of wheat dietary fibre (WF) of varying particle size: WF 500 (fibre length 500 μm) and WF 90 (fibre length 90 μm), which are commercially used in the food industry to give meat products (finely ground cold meats) desirable texture and sensory properties. The material was obtained from “Interfiber” company (Warsaw, Poland), Table 1 (online resource).

### *In vitro* Digestion

The digestive process (30 g of sample) was carried out according to Dziedzic *et al.* [1] with a modification related to the size of the reaction tank (1 L). The environment of the stomach, small and large intestine was reproduced as closely as possible [1, 17]. This paper uses a model which emulates the environment of human gastrointestinal tract with its pH, the presence of enzymes *i.e*., pepsin (0.576 g in 12 mL of 0.1 M sterilized hydrochloric acid), pancreatin (0.12 g) and bile acid salts (cholic acid (CA), DCA, LCA, each 0.36 g)- Sigma-Aldrich, Seelze, Germany, mixed together in 30 mL of 0.1 M sterilized sodium bicarbonate. In order to further enhance the resemblance of this environment to the human digestive tract (large intestine in particular), a mix of faecal bacteria, previously isolated from a healthy 24 year old male, was added at stage 2 (anaerobic conditions), in the amount of 10^4^–10^6^ cfu/mL. A bioreactor (300 mL of total volume) was used as a simple digestive tract, and the samples were collected in three stages of digestion (1 – duodenum, pH 6.0; 2 – ileum, pH 7.2; 3 - colon, pH 8.0). The simulation of the gastrointestinal tract was conducted at 37 °C, in anaerobic conditions and at the stirring speed of 200 rpm.

### Dietary Fibre Assay

#### Total Dietary Fibre (TDF)

The contents of TDF, consisting of soluble dietary fibre (SDF) and insoluble dietary fibre (IDF) were estimated using enzymatic method [18]. The assumption in this method is to determine the content of dietary fibre under conditions similar to those found in the human alimentary tract using the following enzymes: thermostable α-amylase (Termamyl 120 L, pH 6.0, 90°C, 15 min.)- Novozymes, Bagsvaerd, Denmark; pepsin (pH 1.5, 40°C, 1 h) and pancreatin (pH 6.8, 40°C, 1 h)- Sigma-Aldrich, Seelze, Germany. Following the enzymatic extraction, the samples were washed with 3 × 20 mL of hot water, 3 × 10 mL of 96 % ethanol and 3 × 20 mL of acetone (Poch, Gliwice, Poland, pure p.a.). Filters with the residue (IDF) were dried at 135°C for 2 h and then incinerated for 5 h in an oven at 525°C. In order to determine SDF the filtrate was mixed with 96 % ethanol (400 mL, 60°C) and left for 2 h. The precipitated dietary fibre was washed with 3 × 20 mL of hot water, 3 × 10 mL of 96 % ethanol and 3 × 20 mL of acetone, and then the filters with the residue (SDF) were dried at 135°C for 2 h and incinerated in an oven at 525°C for 5 h. Analyses were performed using a Fibertec System 1023 apparatus (Foss, Sweden).

$$ \%IDF\; or\%SDF=\frac{\left(\left( Weight\; of\; residue- protein- ash\right)- blank\right)*100}{weight\; of\; sample} $$

#### Detergent Fibre Determination

The content of neutral dietary fibre (NDF), consisting of acid detergent fibre (ADF) and acid detergent lignin (ADL), was determined using the detergent method [19], as modified by McQueen & Nicholson [20]. Thermostable α-amylase was used to digest starch. Reagents used to estimate the content of neutral detergent fiber (NDF) were: neutral disodium versenate dehydrate, disodium tetraborate decahydrate, disodium hydrogen phosphate, ethylene glycol (Poch, Gliwice, Poland, pure p.a.) and redistilled water. The reagents used to estimate the content of ADF: sulfuric acid (1 N, Poch, Gliwice, Poland, pure p.a.), N-cetyl-N,N,N- trimethylammonium bromide (Merck, Darmstadt, Germany, GR for analysis). The reagent used to estimate the content of ADL: sulfuric acid (72 %, Poch, Gliwice, Poland, pure p.a.). Hemicellulose (H) content was calculated from the difference between NDF and ADF. Cellulose (C) content was calculated from the difference between ADF and ADL. Analyses were conducted using a Fibertec System M 1020 apparatus by Tecator (Foss, Sweden).

$$ \%\mathrm{Hemicellulose}=\%NDF-\%ADF $$

$$ \%\mathrm{Cellulose}=\%ADF-\%ADL $$

### Bile Acid Assay

Bile acids (CA, DCA and LCA) were analysed using LC-MS method described by Dziedzic et al. [1]. Ultra high-performance liquid chromatography electrospray ionization mass spectrometry analysis was performed using a DionexUltiMate 3000 UHPLC (Thermo Fisher scientific, CA, USA) coupled with a Bruker maXis impact ultrahigh resolution orthogonal quadrupole-time-of-light accelerator (qTOF) equipped with an ESI source and operated in the positive-ion Dean distance measure.

#### Content of Faecal Bacteria

Microbiological research was carried out according to international standards [21]. The count of *Enterococcus*, *Bifidobacterium*, *E. coli* and *Lactobacillus* in the experimental samples was determined using the general pour plate technique on Kanamycine Esculine Azide Agar for *Enterococcus* spp., TOS agar with MUP Selective Supplement for *Bifidobacterium* spp., Endo agar for *E. coli* spp., and MRS agar for *Lactobacillus* spp*.* (Merck, Darmstadt, Germany).

#### Short-Chain Fatty Acid Assay

Determination of organic acids (acetic acid, propionic acid, lactic acid, butyric acid) was carried out using UHPLC (VWR-HITACHI LaChrom Elite) system consisting of an autosampler (model L-2200), pump (model L-2130) and a UV detector (*L-2400*) connected in a series. Analyses were performed isocratically at a flow rate of 0.6 mL/min at 40 °C, on Rezex ROA - Organic Acid H+, 300 × 7.8 mm (Phenomenex) column. Sulfur acid (0.005 N) as a mobile phase was used. Standards (lactic acid- 1.1, 0.55, 0.275, 0.11 g/L; acetic acid- 1.0, 0.5, 0.25, 0.1 g/L; propionic acid- 1.0, 0.5, 0.25, 0.1 g/L and butyric acid- 0.55, 0.275, 0.1375, 0.055 g/L; Sigma-Aldrich, Seelze, Germany) were used to identify peaks in chromatograms, and peak area was used to determine the concentration of samples. It was done by computer integration (EzChrom Elite, Version 3.3.2 SP2) operated in the mode of external standard [22].

### Statistical Analysis

Experiments were conducted with three replications. Each value was the mean of three independent trials (Tables 2 and 2, online resource). One-way analysis of variance (ANOVA) was performed. Hierarchical cluster analysis was carried out using Ward amalgamation rule with the Euclidean distance (*d*) measure*.* Tree plots were scaled to a standardized scale (*dlink*/dmax*100). Non-hierarchical cluster analysis (*k*-means clustering) was performed to form a grouping of wheat fibre samples. V-fold cross-validation algorithm was used to determine the best number of clusters. Principal component analysis (PCA) technique was used to reduce the dimensionality of data and to present the samples in a new coordinate system. Statistica software, Version 10, StatSoft Inc. (OK, USA) was used to carry out statistical analysis.

## Results and Discussion

**Fig. 1:**
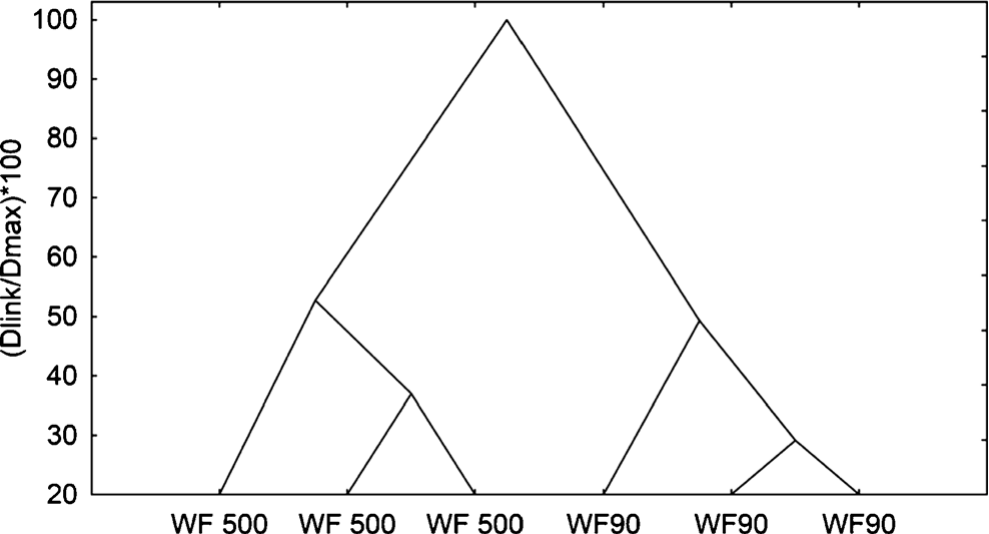
The results of the cluster analysis showing the variation within content of dietary fibre, lipids, proteins and ash in wheat fiber (WF 90, WF 500). The normalisation of scale tree to dlink/dmax*100 was performed (d – distance, l – linkage, max – maximum of linkage Euclidean distance). Amalgamation rule: Ward’s method, distance metric: Euclidean distances

**Fig. 2:**
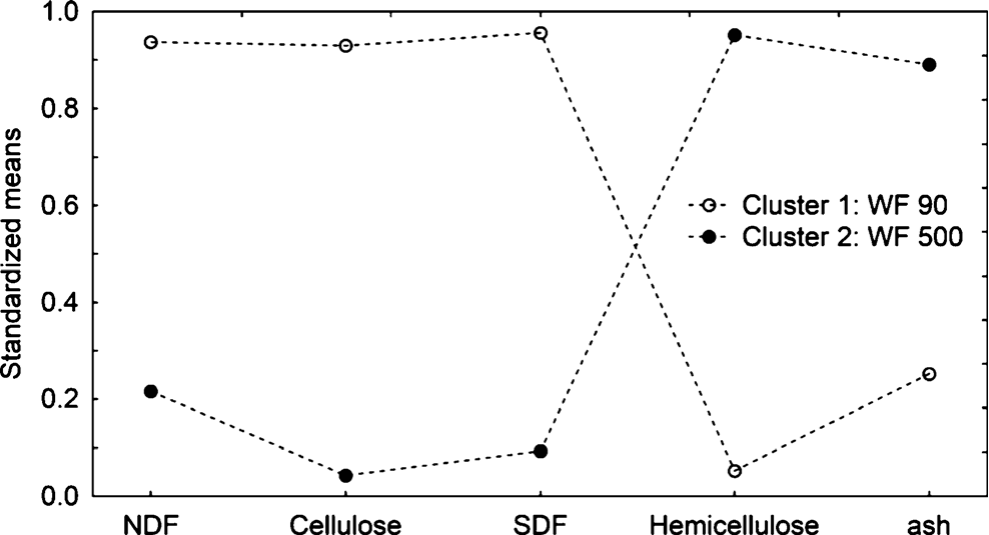
The results of k-means clustering obtained two groups showing radically different means for dietary fibre fractions and ash in wheat fiber samples (WF 90, WF 500). Presented profile was generated for variables with discriminant power (*p* < 0.05) in k-mean procedure

The differences in the content of dietary fibre, ash, protein and lipids in wheat fibre (WF 90 and WF 500) samples were investigated. The analysis of hierarchical tree showed that WF 90 and WF 500 profiles are significantly different (Fig. 1). The k-means algorithm was used to detect differences between wheat fibre samples. The ANOVA (Analysis of variance) results pertaining to the differences in the means for the continuous variables across the clusters were used to evaluate which variables had discriminant power (*p* < 0.05) - Table 4 (online resource). WF 90 and WF 500 were distinguished by the content of ash, NDF, C, H, ligning (L) and SDF. The line graph (Fig. 2) shows the scaled cluster means for continuous variables. The plotted values depict the means for cluster 1 (WF 90) and 2 (WF 500) generated with k-means algorithm. The means of variables for cluster 1 are the opposite of means for cluster 2. Coda *et al.* [23] demonstrated the influence of oat bran fragmentation on the content of SDF and IDF fractions of fibre. Oat bran of greater fineness (50 μm) contained more soluble fraction (SDF) than less fragmented oat bran (750 μm). Zhu *et al.* [24] established the influence of oat bran fragmentation on lowering the TDF content while increasing the SDF fraction content. The degree of fineness also determines the functional properties of dietary fibre. It was documented that particle size of dietary fibre plays an important role in transit time, fermentation and faecal excretion. A reduction in the particle size of coconut fibre resulted in improved hydration properties. The fat absorption capacity was also shown to increase with the decrease in particle size [25].

**Fig. 3:**
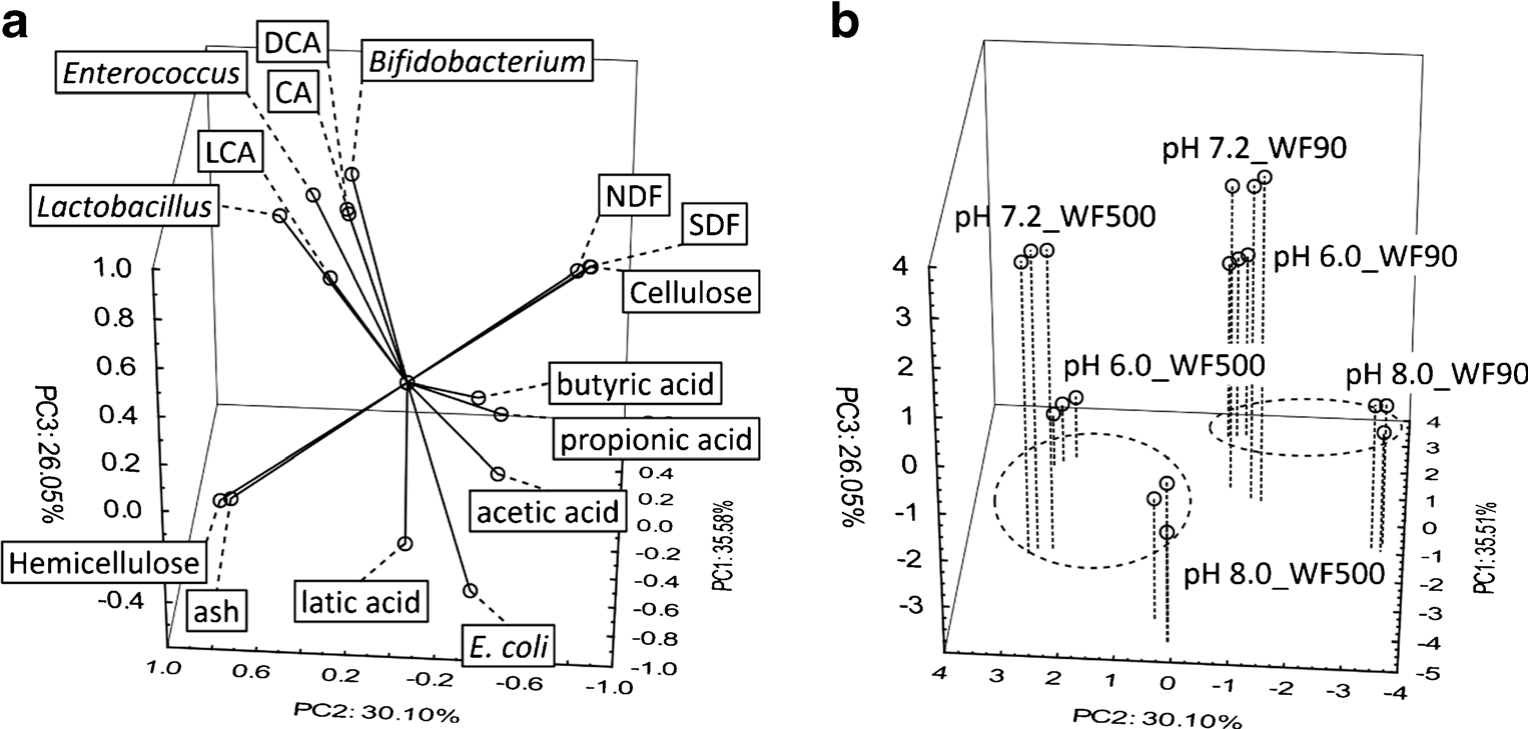
The wheat fiber samples (WF 90 and WF 500) at the three stages of digestion (pH: 6.0, 7.2 and 8.0) were presented in plot representing the PCA of loadings plot **a** where position of vectors indicate the mutual relation between the variables and score plot **b** where the projection of the data into the PCs in three dimensions was involved (PC1, PC2, PC3 – principal components)

PCA was used to explain and interpret interdependences between variables and their impact on the classification of data. Variables with discriminant power for wheat fibre samples (computed using k-Means procedure) in PCA analysis were used. In this study, PCA was used to reduce the number of variables of the dataset (characteristics of WF samples, profile of bile acid content, concentration of organic acids and bacteria at three stages of digestion). The plot of principal component (PC) coordinates of variables for the first three factors (PCs) made the interpretation of principal components much easier – Fig. 3a (*x*-loadings plot). Three new variables (PCs) were introduced. Component 1 had the highest explanatory power, while components 2 and 3 together explained about 92 % of the data variance for the three stages of digestion (pH 6.0, 7.2 and 8.0). As shown in Fig. 3B, it is possible to distinguish two groups. The first cluster represents WF 90 and the second cluster represents WF 500. It was indicated that parameters for the two groups were significantly different. The simultaneous comparison loading plot (Fig. 3a) with appropriate score plot (Fig. 3b) allowed to identify the relationships between samples and variables.

The results of PCA analysis led to the conclusion that WF 90 and WF 500 form two distinct groups, characterized by the qualitative composition of dietary fibre. The first group is described by a high content of NDF, SDF and cellulose, typical for WF 90. The second group consists of samples with WF 500 dietary fibre, characterized by a high content of hemicellulose and ash. The research conducted by Silva *et al.* [26] demonstrated a reduction in the level of NDF in ground hay together with the reduction of particle size, therefore, the hemicellulose fraction content was higher in ground hay of larger particle size. Because vectors depicting such variables as hemicellulose and ash point in the opposite directions to those representing NDF, SDF and cellulose, it can be concluded that these variables are inversely correlated. Analogous sample profile was obtained for the three stages of digestion (pH – 6.0, 7.2 and 8.0) in each analysed group. WF 90 and WF 500 are situated within each group depending on the digestive stage, which had a direct bearing on the content of bile acids, fatty acids, and the growth intensity of the observed microorganisms. An increase in the growth of *Lactobacillus* spp., *Enterococcus* spp. and *Bifidobacterium* spp. bacteria was observed during the process of digestion, with their count reaching a maximum at pH 7.2. Further incubation, accompanied by the rise in pH to 8 in the third phase, resulted in a reduction in the numbers of the above mentioned bacteria, while at the same time causing a significant growth in *E. coli* spp. This change of proportions between the various groups of microorganisms can be attributed not only to the changes in the growth conditions, but also to the interspecific competition; the latter can be suggested by the interrelation of variables in Fig. 3a. In the third phase of digestion (pH 8), both the *E. coli* spp. count and the concentration of short-chain fatty acids was the highest. High concentration of bile acids was strongly correlated with the high count of *Lactobacillus* spp., *Enterococcus* spp. and *Bifidobacterium* spp., while the lowest values were noted at pH 6.0 and 8.0. *Bifidobacterium* spp. have the ability to synthesize hydrolases, which are responsible for the transformation of primary bile acids such as CA to secondary bile acids such as DCA and LCA [6]. This can explain the strong correlation between CA, DCA and *Bifidobacterium* spp. vectors. In previous research, Dziedzic *et al.* [1] observed that digestive tract microflora can be responsible for the creation of primary bile acids. Research by Pereira *et al.* [27] demonstrated the presence of enzymatic proteins similar to those identified in the liver, which is attributed to the synthesis of primary bile acids from cholesterol - cytochrome P450 proteins. However, in this research no cholesterol was applied. Therefore a higher concentration of bile acids at stage 2 could be explained by their better solubility in pH 7.2, as compared with stage 1 (pH 6.0). Hofmann and Mysels [28] suggested that the pH of the environment affects the solubility of bile acids and the ability to form soluble salts. Neither the degree of fibre fineness, nor its basic composition influenced the concentration of bile acids and the growth of bacteria (their vectors are orthogonal to the vectors depicting fibre composition). However, a higher content of NDF, SDF and cellulose in WF 90 fiber promoted the synthesis of butyric, propionic and acetic acids. All this leads to the conclusion that the type of fibre used had no effect on the concentration of bile acids at any of the three stages of digestion. An increase in the content of SCFA during the digestive process including WF 90 fibre can be explained by its influence on the expression of genes responsible for the synthesis of SCFA [29]. Special attention should be paid to the significant increase in *E. coli* spp. count, a fall in the concentration of bile acids and a rise in the concentration of short-chain fatty acids at the final stage of digestion (pH 8.0). *Bifidobacterium* spp. and *Lactobacillus* spp. bacteria demonstrated an antagonistic effect on *E. coli* bacteria [30]. It should be emphasized that these bacteria are too sensitive to pH (4.0–5.0, 7.5–8.0) and require a neutral environment with pH in the range 6.0–7.0 [31]. It has also been suggested that a longer exposition of these bacteria to the environment containing bile acids results in the adhesion of these acids to the membranes of bacterial cell walls and entering into the cells. Bile acids can then modify in various ways the expression of bacterial DNA leading to a fall in the numbers of bacteria. *E. coli* spp. are less sensitive to changing pH conditions, and they have a mechanism for eliminating bile acids from cell membranes, therefore, their count at the last stage of digestion (pH 8.0) was observed to rise [32].

## Conclusions

During digestion of fibre fractions of greater fineness (WF 90) an increase in the content of propionic and butyric acids was observed, as compared with fibre fraction WF 500. Moreover, the effect of bile acid concentration on the growth of *Bifidobacterium* spp. and *Lactobacillus* spp. was demonstrated. Lactic acid bacteria (LAB) bind bile acids and exhibit an antagonistic effect towards *E. coli* spp. bacteria in the presence of dietary fibre of varied fineness. A growth in the population of *E. coli* spp. was accompanied by a decrease in the numbers of *Bifidobacterium* spp. and *Lactobacillus* spp. with a simultaneous reduction in the concentration of bile acids. This strongly suggests a direct binding influence of LAB on CA, LCA and DCA acids. The level of fineness of wheat dietary fibre does not affect the concentration of bile acids and the growth of bacteria; however, its influence on the profile of SCFA synthesized by bacteria was demonstrated.

## Electronic supplementary material

ESM 1 (PDF 55 kb)

ESM 2 (PDF 106 kb)

ESM 3 (PDF 104 kb)

ESM 4 (PDF 13 kb)

## Abbreviations

C: cellulose
CA: cholic acid
D: distance
DCA: deoxycholic acid
H: hemicellulose
IDF: insoluble dietary fibre
L: lignin
l: linkage
LCA: lithocholic acid
Max: maximum of linkage Euclidean distance
NDF: neutral detergent fibre
PC: principal component
SCFA: short-chain fatty acids
SDF: soluble dietary fibre
TDF: total dietary fibre
UHPLC: ultra high performance liquid chromatography
WF: wheat fibre
