# Supplementary material for: Effect of Wheat Dietary Fiber Particle Size during Digestion In Vitro on Bile Acid, Faecal Bacteria and Short-Chain Fatty Acid Content
Source: Plant Foods Hum Nutr. 2016 Feb 29;71:151–7. doi: 10.1007/s11130-016-0537-6 (PMC4891393; doi:10.1007/s11130-016-0537-6)
Supplement: Supplementary file 1 — (PDF 55 kb) [file 11130_2016_537_MOESM1_ESM.pdf]

Tab 1 The characteristic of wheat fiber (mg/100 g of product ± SD)

	dm	NDF	C	H	L	TDF	IDF	SDF
WF 90	97.27±0.31	99.33±0.06	89.02±0.54	9.86±0.46	0.22±0.02	90.53±0.55	89.66±0.53	0.87±0.03
WF 500	95.83±0.41	98.63±0.30	81.15±0.42	17.25±0.54	0.23±0.05	89.94±0.45	89.71±0.51	0.23±0.05

Abbreviations: dm- dry matter; NDF- neutral detergent fiber; C- cellulose; H- hemicellulose; L- lignin; TDF- total dietary fiber; IDF- insoluble dietary fiber; SDF- soluble dietary fiber
