# Supplementary material for: Effect of Wheat Dietary Fiber Particle Size during Digestion In Vitro on Bile Acid, Faecal Bacteria and Short-Chain Fatty Acid Content
Source: Plant Foods Hum Nutr. 2016 Feb 29;71:151–7. doi: 10.1007/s11130-016-0537-6 (PMC4891393; doi:10.1007/s11130-016-0537-6)
Supplement: Supplementary file 2 — (PDF 106 kb) [file 11130_2016_537_MOESM2_ESM.pdf]

Tab 2 The content of faecal bacteria and organic acid during *in vitro* digestion

	E.coli	*Enterococcus*	*Lactobacillus*	*Bifidobacterium*	lactic a.	acetic a.	propionic a.	butyric a.
	(log cfu/mL ± SD)				(g/L ± SD)			
6.0_WF 90	6.36±0.13	6.32±0.15	6.44±0.06	0.00±0.00	0.13±0.00	0.05±0.00	0.1±0.00	0.04±0.00
6.0_WF 500	6.58±0.26	6.39±0.19	6.50±0.07	0.00±0.00	0.11±0.01	0.05±0.00	0.08±0.00	0.03±0.00
7.2_WF 90	6.36±0.13	6.31±0.15	6.46±0.06	3.62±0.03	0.30±0.03	0.17±0.02	0.66±0.07	0.05±0.01
7.2_WF 500	6.61±0.25	6.39±0.19	6.50±0.07	3.36±0.32	0.33±0.01	0.16±0.00	0.57±0.00	0.06±0.00
8.0_WF 90	7.42±0.18	4.79±0.29	5.32±0.39	0.00±0.00	0.39±0.02	0.25±0.01	0.81±0.04	0.06±0.00
8.0_WF 500	7.57±0.23	4.46±0.20	5.67±0.12	0.00±0.00	1.51±0.02	0.26±0.01	0.72±0.01	0.06±0.00

Abbreviations: 6.0_WF 90- wheat fiber, 90 µm, pH 6.0; 6.0_WF 500- wheat fiber, 500 µm, pH 6.0; 7.2_WF 90- wheat fiber, 90 µm, pH 7.2; 7.2_WF 500- wheat fiber, 500µm, pH 7.2; 8_WF 90- wheat fiber 90 µm, pH 7.2; 8_WF 500- wheat fiber 500 µm, pH 8.0; a.- acid
