# Supplementary material for: Effect of Wheat Dietary Fiber Particle Size during Digestion In Vitro on Bile Acid, Faecal Bacteria and Short-Chain Fatty Acid Content
Source: Plant Foods Hum Nutr. 2016 Feb 29;71:151–7. doi: 10.1007/s11130-016-0537-6 (PMC4891393; doi:10.1007/s11130-016-0537-6)
Supplement: Supplementary file 3 — (PDF 104 kb) [file 11130_2016_537_MOESM3_ESM.pdf]

Tab 3 The content of bile acid during *in vitro* digestion

	pH	CA	DCA	LHA	ash	NDF	C	H	SDF
	(mg/mL of sample ± SD)				(g/100 g of product ± SD)				
6_WF 90	6.00	26.9±0.34	11.16±0.12	0.28±0.00	0.30±0.02	99.33±0.06	89.02±0.54	9.86±0.46	0.87±0.03
6_WF 500	6.00	11.94±0.50	0.79±0.02	0.06±0.01	0.36±0.01	98.63±0.30	81.15±0.42	17.25±0.54	0.23±0.07
7.2_WF 90	7.20	29.76±0.36	25.58±0.42	1.82±0.03	0.30±0.02	99.33±0.06	89.02±0.54	9.86±0.46	0.87±0.03
7.2_WF 500	7.20	35.58±1.08	31.30±0.11	3.24±0.01	0.36±0.01	98.63±0.30	81.15±0.42	17.25±0.54	0.23±0.07
8_WF 90	8.00	14.84±0.14	5.75±0.01	0.82±0.01	0.30±0.02	99.33±0.06	89.02±0.54	9.86±0.46	0.87±0.03
8_WF 500	8.00	26.35±1.08	15.89±0.96	1.55±0.04	0.36±0.01	98.63±0.30	81.15±0.42	17.25±0.54	0.23±0.07

Abbreviations: see Supplemental Table 2; CA- cholic acid; DCA- deoxycholic acid; LHA- lithocholic acid; NDF- neutral detergent fiber; C- cellulose; H- hemicellulose; SDF- soluble dietary fiber
