# Supplementary material for: Effect of Wheat Dietary Fiber Particle Size during Digestion In Vitro on Bile Acid, Faecal Bacteria and Short-Chain Fatty Acid Content
Source: Plant Foods Hum Nutr. 2016 Feb 29;71:151–7. doi: 10.1007/s11130-016-0537-6 (PMC4891393; doi:10.1007/s11130-016-0537-6)
Supplement: Supplementary file 4 — (PDF 13 kb) [file 11130_2016_537_MOESM4_ESM.pdf]

Tab 4 Variation in the composition of wheat fiber (WF 90, WF 500), expressed as percentages of dry matter and ANOVA results, evaluation of discriminant power of variables ($p<0.05$) in k-Mean clustering procedure

Variable	WF90 (%)	WF 500 (%)	Between - SS	df	Within - SS	df	F	p value
Lipids	0.09	0.12	0.838	1	4.162	4	0.806	0.4201
Protein	0.03	0.03	0.000	1	5.000	4	0.000	0.9983
Ash	0.30	0.36	4.133	1	0.867	4	19.061	0.0120
NDF	99.33	98.63	3.990	1	1.010	4	15.800	0.0165
Cellulose	89.02	81.15	4.949	1	0.051	4	391.395	0.0000
Hemicellulose	9.86	17.25	4.939	1	0.061	4	323.940	0.0001
Lignin	0.22	0.23	0.283	1	4.717	4	0.240	0.6501
SDF	0.87	0.23	4.918	1	0.082	4	240.989	0.0001
IDF	89.66	89.71	0.018	1	4.982	4	0.014	0.9111
TDF	90.53	89.94	1.722	1	3.278	4	2.102	0.2207
